# Assessing the association between all-cause mortality and multiple aspects of individual social capital among the older Japanese

**DOI:** 10.1186/1471-2458-11-499

**Published:** 2011-06-25

**Authors:** Jun Aida, Katsunori Kondo, Hiroshi Hirai, S V Subramanian, Chiyoe Murata, Naoki Kondo, Yukinobu Ichida, Kokoro Shirai, Ken Osaka

**Affiliations:** 1 Department of Epidemiology and Public Health, University College London, London, UK; 2 Department of International and Community Oral Health, Tohoku University Graduate School of Dentistry, Sendai, Japan; 3 Center for Well-being and Society, Nihon Fukushi University, Nagoya, Japan; 4 Department of Society, Human Development, and Health, Harvard School of Public Health, Boston, USA; 5 Department of Community Health and Preventive Medicine, Hamamatsu University School of Medicine, Hamamatsu, Japan; 6 Department of Health Sciences, Interdisciplinary Graduate School of Medicine and Engineering, University of the Yamanashi, Chuo-shi, Japan; 7 University of the Ryukyus, Naha, Japan

## Abstract

**Background:**

Few prospective cohort studies have assessed the association between social capital and mortality. The studies were conducted only in Western countries and did not use the same social capital indicators. The present prospective cohort study aimed to examine the relationships between various forms of individual social capital and all-cause mortality in Japan.

**Methods:**

Self-administered questionnaires were mailed to subjects in the Aichi Gerontological Evaluation Study (AGES) Project in 2003. Mortality data from 2003 to 2008 were analyzed for 14,668 respondents. Both cognitive and structural components of individual social capital were collected: 8 for cognitive social capital (trust, 3; social support, 3; reciprocity, 2) and 9 for structural social capital (social network). Cox proportional hazard models stratified by sex with multiple imputation were used. Age, body mass index, self-rated health, current illness, smoking history, alcohol consumption, exercise, equivalent income and education were used as covariates.

**Results:**

During 27,571 person-years of follow-up for men and 29,561 person-years of follow-up for women, 790 deaths in men and 424 in women were observed. In the univariate analyses for men, lower social capital was significantly related to higher mortality in one general trust variable, all generalised reciprocity variables and four social network variables. For women, lower social capital was significantly related to higher mortality in all generalised reciprocity and four social network variables. After adjusting for covariates, lower friendship network was significantly associated with higher all-cause mortality among men (meet friends rarely; HR = 1.30, 95%CI = 1.10-1.53) and women (having no friends; HR = 1.81, 95%CI = 1.02-3.23). Among women, lower general trust was significantly related to lower mortality (most people cannot be trusted; HR = 0.65, 95%CI = 0.45-0.96).

**Conclusions:**

Friendship network was a good predictor for all-cause mortality among older Japanese. In contrast, mistrust was associated with lower mortality among women. Studies with social capital indices considering different culture backgrounds are needed.

## Background

Few prospective cohort studies have assessed the association between social capital and mortality [1-5]. The studies did not use the same social capital indicators [1-5]. Some of these studies used proxy measures of social capital [6,7], such as crime rate [1], electoral participation [1,5] or volunteer activity [3-5]. There are several components of social capital, such as social network, participation, trust, reciprocity and volunteering [8]. Previous studies on social capital and mortality did not simultaneously use various components of social capital and their results were not fully consistent. In Finland, the association between mortality and individual social capital variables obtained by factor analysis (leisure participation, interpersonal trust and residential stability) was examined [2]. In men, leisure participation was associated with reduced all-cause mortality. In women, leisure participation and interpersonal trust were associated with reduced all-cause mortality. In a Swedish study, survival analyses showed that both neighbourhood social capital variables (election participation rate and crime rate) were significantly associated with mortality for males older than 65 years old but not for females [1]. Another study showed that living in a neighbourhood with the lowest level of social capital (volunteering, participation, political activities) was associated with significantly higher mortality than living in a neighbourhood with the highest level of social capital in England [5]. In contrast, among adults diagnosed and hospitalized with serious illnesses in the U.S, neighbourhood social capital (network density) was detrimental [4]. In addition, other neighbour social capital variables (social support, participation, volunteering, violence) did not significantly affect mortality [4]. In New Zealand, non-significant associations between neighbourhood social capital (volunteering) and mortality for both male and female were observed [3].

Studies on mortality and social capital have been conducted only in Western countries. However, social capital measurements developed in Western countries may not necessarily be equally applicable to Asian countries because of their different culture [9]. Although general trust has been broadly used as a measurement of social capital [10], it is known that intense ties within a family or group, often observed in collectivist cultures, prevent trust from developing beyond family or group boundaries [11-13]. In Japan, a relatively collectivist society with intense group ties, human relationships are based on mutual assurance within group members rather than mutual trust between members from different groups [11,13]. These cultural differences could potentially affect findings on the associations between social capital and health outcomes. In this respect, a social epidemiological study using various social capital indices in a non-Western cultural setting is important.

There is still debate about the precise definition and measurement of social capital [8,14,15]. Bourdieu defined social capital as "the aggregate of the actual or potential resources which are linked to possession of a durable network of more or less institutionalised relationships of mutual acquaintance and recognition"[16] and which focuses on the resources of individuals [8]. It is important to determine the association between individual social capital and health, because individual social capital indexes are components of aggregated measurements of community social capital [17]. Additionally, individual measures of social capital are not subject to the common problems arising from using area measurement in epidemiological studies, such as definition of a relevant areas [18,19]. No study has used various measures of individual social capital as a predictor of mortality in a non-Western country. The aim of the present prospective cohort study was to assess the influence of individual social capital on all-cause mortality among older Japanese.

## Methods

### Study population and procedure

The present analysis is based on the Aichi Gerontological Evaluation Study (AGES) Project data, an on-going prospective cohort study [20-24]. AGES investigates factors associated with the loss of health, including death and functional decline or cognitive impairment among older individuals. The study was undertaken in six municipalities covered the entire southern part of the Chita peninsula in Aichi Prefecture, Japan. During October one to 31 2003, a baseline mail questionnaire survey was administered. The follow-up started in November one 2003. Mortality data until May 2008 were obtained from 6 of the municipalities participating in AGES.

**Figure 1:**
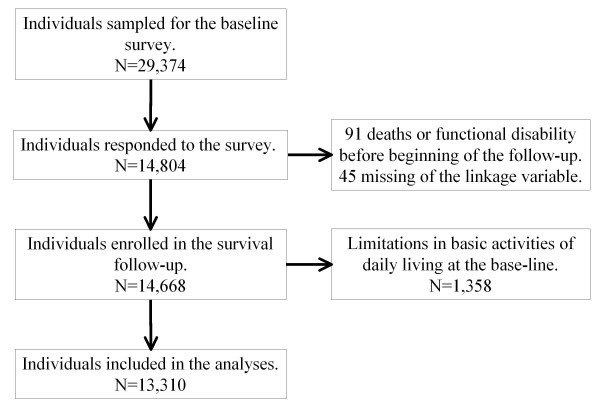
**Flowchart of the Aichi Gerontological Evaluation Study (AGES), Aichi, Japan, 2003-2008**.

In 2003, there were 274,750 people living in the six municipalities, 17.9% of them being 65 years or older. The sample was restricted to people who did not already have physical or cognitive disabilities, defined as receiving public long-term care insurance benefits. From the municipalities, 29,374 community-dwelling, aged 65 years or over people were selected randomly. From this sample population, 14,804 people responded to the baseline survey. Of the 14,804 respondents, we could link the mortality data and baseline survey data on 14,668 subjects, because 91 were ineligible due to death, functional decline or cognitive impairment before November 1, 2003, and for a further 45 there was no information that would allow linking of the mortality data. Some subjects did not apply to certification of long-term care needs though they had limitations in basic activities of daily living including walking, bathing and toilet use. We excluded them from the analysis to avoid potential confounding (1,358 of the 14,668 respondents). Finally, 13,310 subjects (6,508 men and 6,802 women.) were included in the analysis for this cohort study. Figure 1 shows the study profile. Characteristics of participants at baseline have been reported elsewhere [23,24]. The AGES protocol was reviewed and approved by the ethics committee in Research of Human Subjects at Nihon Fukushi University.

### Social capital variables

Both cognitive and structural components [8,10,17,25] of individual social capital were used. We basically followed Harpham's classification of social capital [17]. We used eight cognitive social capital variables, including general trust, social support and generalised reciprocity, and nine structural social capital variables, including social networks.

### Cognitive social capital

General trust was measured by 3 questions: "Generally speaking, would you say that most people can be trusted?", "Do you think most people would try to take advantage of you if they got a chance?" and "Would you say that most of the time people try to be helpful?". For all these questions, response alternatives were "yes", "it depends" and "no".

Social support was measured by three questions, using a dichotomous answering choice (yes/no): "Do you have someone who listens to your concerns and complaints?", "Do you have someone who looks after you when you are sick and stay in bed for a few days?" and "Do you have someone who acknowledges your existence and value?".

Generalised reciprocity was measured by two questions, again with a dichotomous choice (yes/no): "Do you listen to someone's concerns and complaints?" and "Do you look after someone when he/she is sick and stays in bed for a few days?".

### Structural social capital

Participation in community activities was used as an indication of social network. Respondents were asked whether they belonged to a (i) political organization or group, (ii) industrial or trade association, (iii) volunteer group, (iv) citizen or consumer group, (v) religious organization or group, (vi) sports group or club, (vii) neighbourhood association / senior citizen club / fire-fighting team and (viii) leisure activity group.

Social network was also measured by the question "How often do you meet your friends?" (response options: "almost everyday", "twice or three times a week", "once a week", "once or twice a month", "several times a year", "rarely" or "I have no friends"). For analyses purposes, the first four response options were integrated into one category, named "once or more/month".

### Covariates

We also asked about socio-demographic characteristics, lifestyle and health condition and included the following in the analyses as covariates: age, sex, self-reported body mass index (BMI), self-rated health, present illness, smoking history, alcohol consumption, exercise, equivalent income and educational attainment [23]. Self-reported BMI was categorized into 4 groups (less than 18.5, 18.5-24.9, 25-29.9, 30 or more). Self-rated health was measured by a single question, "What is your current health status?: Excellent; Good; Fair; Poor". Present illnesses and present medical treatment were surveyed as follows: "Are you currently receiving any medical treatment?: I have no illnesses or disabilities; I have illness(es) or disability(ies) but need no treatment at the moment; I discontinued treatment of my own decision; I am currently receiving some treatment. Smoking history was recorded in 3 categories (never, quit or current) and alcohol consumption into 4 categories (non-drinker, do not drinking everyday, drinking 35 g of alcohol or less daily, or drink more than 35 g every day). Subjects were asked about how many minutes a day they walk - the exercise variable; less than 30 minutes, 30-60, 60-90 or more than 90 minutes or more. Years of educational attainment was grouped as less than 6 years, 6-9 years, 10-12 years and 13 years or more. Household income and number of household members were recorded and then equivalent income was calculated and categorized in Yen: less than 1,500,000; 1,500,000-1,999,999; 2,000,000-2,499,999; 2,500,000-2,999,999; 3,000,000-3,499,999; 3,500,000-3,999,999; 4,000,000-4,999,999; 500,000,000 or higher.

### Mortality outcome

Mortality obtained from the municipality government registry was treated as all-causes.

### Analysis

We used Cox proportional hazard models to calculate the hazard ratio (HR) and 95% confidential intervals (95%CI) for all-cause mortality during the follow-up period. At first, we calculated univariate hazard ratios for mortality for the categories of each social capital variable. In the covariate adjusted models, we assessed the effect of each social capital variable on mortality with adjustment for age, BMI, self-rated health, current illness, smoking history, alcohol consumption, exercise, equivalent income and educational attainment. All analyses were stratified by sex.

In terms of analysis of missing data (numbers of missing responses in each variable are described in Table 1,2), we used the missing at random assumption for the relevant procedures. Multiple imputation with the MICE (multivariate imputation by chained equations) method in STATA was used [26]. Cox proportional hazard models were independently applied for 10 copies of the data, each with missing values suitably imputed. Estimates of the variables were calculated to give a single mean estimate and adjusted standard errors according to Rubin's rules [27]. HRs and 95%CI of the Cox proportional hazard models were calculated from these estimates. We show results both from multiple imputation analyses and analyses with complete data for each model (non-imputation analyses). STATA SE version 11.1 (Stata Corp, College Station, TX) was used and sample weights were applied when estimating HR.

**Table 1:** Characteristics of the subjects by mortality rate: the Aichi Gerontological Evaluation Study (AGES), Aichi, Japan, 2003-2008

	Man			Woman		
	N	Incidence/person-year	Incidence rate (95% CI) (1000 person-years)	N	Incidence/person-year	Incidence rate (95% CI) (1000 person-years)
Age						
65-69	2528	164/10978	14.9 (12.8-17.4)	2325	62/10285	6.0 (4.7-7.7)
70-74	1982	181/8524	21.2 (18.4-24.6)	1908	76/8377	9.1 (7.2-11.4)
75-79	1272	214/5288	40.5 (35.4-46.3)	1510	102/6533	15.6 (12.9-19.0)
80-84	516	138/2035	67.8 (57.4-80.1)	715	82/3029	27.1 (21.8-33.6)
85 or older	210	93/746	124.6 (101.7-152.7)	344	102/1336	76.3 (62.9-92.7)
Education (years)						
<6	154	31/628	49.3 (34.7-70.1)	407	60/1710	35.1 (27.2-45.2)
6-9	3322	441/14046	31.4 (28.6-34.5)	3695	212/16108	13.2 (11.5-15.1)
10-12	1755	179/7493	23.9 (20.6-27.7)	1959	109/8523	12.8 (10.6-15.4)
≥13	885	83/3764	22.1 (17.8-27.3)	343	15/1487	10.1 (6.1-16.7)
Missing	392	56/1641	34.1 (26.3-44.3)	398	28/1733	16.2 (11.2-23.4)
Individual-level equivalent income ($)						
<15,000	1111	166/4634	35.8 (30.8-41.7)	1361	88/5889	14.9 (12.1-18.4)
15,000-19,999	1132	116/4826	24.0 (20.0-28.8)	812	38/3563	10.7 (7.8-14.7)
20,000-24,999	1364	171/5751	29.7 (25.6-34.5)	949	49/4155	11.8 (8.9-15.6)
25,000-29,999	337	43/1415	30.4 (22.5-41.0)	295	19/1276	14.9 (9.5-23.3)
30,000-39,999	1089	103/4695	21.9 (18.1-26.6)	804	42/3505	12.0 (8.9-16.2)
40,000-49,999	382	28/1670	16.8 (11.6-24.3)	367	33/1573	21.0 (14.9-29.5)
≥50,000	278	25/1202	20.8 (14.1-30.8)	220	13/942	13.8 (8.0-23.8)
Missing	815	138/3379	40.8 (34.6-48.3)	1994	142/8657	16.4 (13.9-19.3)
Self-rated health						
Very good	562	41/2440	16.8 (12.4-22.8)	489	22/2121	10.4 (6.8-15.8)
Good	4161	377/17902	21.1 (19.0-23.3)	4367	224/19099	11.7 (10.3-13.4)
Poor	1416	257/5842	44.0 (38.9-49.7)	1541	133/6633	20.0 (16.9-23.8)
Very poor	300	99/1121	88.3 (72.5-107.6)	268	35/1112	31.5 (22.6-43.8)
Missing	69	16/267	60.0 (36.8-98.0)	137	10/595	16.8 (9.0-31.2)
Self-reported BMI						
<18.5	464	116/1835	63.2 (52.7-75.9)	553	70/2333	30.0 (23.7-37.9)
18.5-24.9	4527	511/19269	26.5 (24.3-28.9)	4378	240/19067	12.6 (11.1-14.3)
25-29.9	1249	106/5388	19.7 (16.3-23.8)	1372	63/6028	10.5 (8.2-13.4)
≥30	74	8/317	25.2 (12.6-50.5)	142	5/631	7.9 (3.3-19.0)
Missing	194	49/764	64.2 (48.5-84.9)	357	46/1503	30.6 (22.9-40.9)
Present illness						
No illness	1155	84/5015	16.7 (13.5-20.7)	1056	43/4632	9.3 (6.9-12.5)
Having illness but need no treatment	743	82/3179	25.8 (20.8-32.0)	547	33/2378	13.9 (9.9-19.5)
Having illness but discontinued treatment	404	51/1701	30.0 (22.8-39.4)	448	18/1965	9.2 (5.8-14.5)
Receiving some treatment	3957	545/16599	32.8 (30.2-35.7)	4337	304/18780	16.2 (14.5-18.1)
Missing	249	28/1076	26.0 (18.0-37.7)	414	26/1806	14.4 (9.8-21.1)
Alcohol consumption						
None	2787	433/11555	37.5 (34.1-41.2)	5791	369/25173	14.7 (13.2-16.2)
Do not drink everyday	1156	110/4973	22.1 (18.3-26.7)	589	31/2563	12.1 (8.5-17.2)
Drink every day (35 g of alcohol or less)	1885	175/8121	21.6 (18.6-25.0)	235	10/1018	9.8 (5.3-18.3)
Drink every day (more than 35 g of alcohol)	572	51/2482	20.6 (15.6-27.0)	22	1/92	10.8 (1.5-76.8)
Missing	108	21/442	47.5 (31.0-72.9)	165	13/714	18.2 (10.6-31.4)
Smoking status						
Non-smoker	1772	179/7584	23.6 (20.4-27.3)	6016	355/26191	13.6 (12.2-15.0)
Quit	2991	339/12696	26.7 (24.0-29.7)	271	22/1157	19.0 (12.5-28.9)
Current	1499	220/6297	34.9 (30.6-39.9)	172	20/729	27.4 (17.7-42.5)
Missing	246	52/994	52.3 (39.9-68.6)	343	27/1483	18.2 (12.5-26.5)
Exercise						
Walking less than 30 minutes walk a day	2120	344/8789	39.1 (35.2-43.5)	2176	165/9414	17.5 (15.0-20.4)
Walking 30-60 minutes walk a day	2222	246/9477	26.0 (22.9-29.4)	2101	136/9105	14.9 (12.6-17.7)
Walking 60-90 minutes walk a day	906	74/3908	18.9 (15.1-23.8)	769	25/3378	7.4 (5.0-11.0)
Walking 90 or more minutes walk a day	799	58/3472	16.7 (12.9-21.6)	788	38/3443	11.0 (8.0-15.2)
Missing	461	68/1926	35.3 (27.8-44.8)	968	60/4221	14.2 (11.0-18.3)
Total	6508	790/27572	28.7 (26.7-30.7)	6802	424/29561	14.3 (13.0-15.8)

**Table 2:** Characteristics of the subjects according to social capital and mortality rate: the Aichi Gerontological Evaluation Study (AGES), Aichi, Japan, 2003-2008

		Man			Woman		
		N	Incidence/person-year	Incidence rate (95% CI) (1000 person-years)	N	Incidence/person-year	Incidence rate (95% CI) (1000 person-years)
General trust							
Generally speaking, would you say that most people can be trusted or you cannot be too careful in dealing with people?	Yes (High SC)	2121	252/9007	28.0 (24.7-31.7)	1448	86/6290	13.7 (11.1-16.9)
	Depends	3667	422/15577	27.1 (24.6-29.8)	4480	287/19452	14.8 (13.1-16.6)
	No (Low SC)	545	80/2274	35.2 (28.3-43.8)	667	31/2937	10.6 (7.4-15.0)
	Missing	175	36/713	50.5 (36.4-70.0)	207	20/882	22.7 (14.6-35.1)
Would you say that most of the time people try to be helpful or that they are mostly looking out for themselves?	Yes (High SC)	1954	227/8291	27.4 (24.0-31.2)	1791	95/7817	12.2 (9.9-14.9)
	Depends	3710	412/15796	26.1 (23.7-28.7)	4104	254/17826	14.2 (12.6-16.1)
	No (Low SC)	645	106/2675	39.6 (32.8-47.9)	637	50/2760	18.1 (13.7-23.9)
	Missing	199	45/810	55.5 (41.5-74.4)	270	25/1159	21.6 (14.6-31.9)
Do you think that most people would try to take advantage of you if they got the chance, or would they try to be fair?	Yes (Low SC)	797	101/3377	29.9 (24.6-36.4)	616	40/2689	14.9 (10.9-20.3)
	Depends	3418	378/14536	26.0 (23.5-28.8)	3558	210/15475	13.6 (11.9-15.5)
	No (High SC)	2093	263/8847	29.7 (26.3-33.5)	2318	143/10073	14.2 (12.1-16.7)
	Missing	200	48/812	59.1 (44.5-78.4)	310	31/1323	23.4 (16.5-33.3)
Social support							
Do you have someone who listens to your concerns and complaints?	Yes (High SC)	5267	605/22392	27.0 (24.9-29.3)	5995	360/26087	13.8 (12.4-15.3)
	No (Low SC)	878	122/3671	33.2 (27.8-39.7)	424	34/1819	18.7 (13.4-26.2)
	Missing	363	63/1508	41.8 (32.6-53.5)	383	30/1655	18.1 (12.7-25.9)
Do you have someone who looks after you when you are sick and stay in bed for a few days?	Yes (High SC)	5967	713/25308	28.2 (26.2-30.3)	5988	372/26045	14.3 (12.9-15.8)
	No (Low SC)	258	29/1081	26.8 (18.6-38.6)	485	23/2112	10.9 (7.2-16.4)
	Missing	283	48/1182	40.6 (30.6-53.9)	329	29/1404	20.6 (14.3-29.7)
Do you have someone who acknowledges your existence and value?	Yes (High SC)	5705	664/24243	27.4 (25.4-29.6)	5849	357/25440	14.0 (12.7-15.6)
	No (Low SC)	433	65/1798	36.2 (28.4-46.1)	379	30/1628	18.4 (12.9-26.3)
	Missing	370	61/1532	39.8 (31.0-51.2)	574	37/2492	14.8 (10.8-20.5)
Generalised reciprocity							
Do you listen to someone's concerns and complaints?	Yes (High SC)	4945	522/21122	24.7 (22.7-26.9)	5348	282/23326	12.1 (10.8-13.6)
	No (Low SC)	1153	197/4748	41.5 (36.1-47.7)	941	107/4000	26.8 (22.1-32.3)
	Missing	410	71/1701	41.7 (33.1-52.7)	513	35/2235	15.7 (11.2-21.8)
Do you look after someone when he/she is sick and stays in bed for a few days?	Yes (High SC)	5690	651/24190	26.9 (24.9-29.1)	5785	324/25209	12.9 (11.5-14.3)
	No (Low SC)	461	78/1901	41.0 (32.9-51.2)	526	53/2242	23.6 (18.1-30.9)
	Missing	357	61/1481	41.2 (32.0-52.9)	491	47/2109	22.3 (16.7-29.7)
Social network							
Political group participation	Yes (High SC)	665	72/2848	25.3 (20.1-31.9)	287	13/1250	10.4 (6.0-17.9)
	No (Low SC)	5230	605/22171	27.3 (25.2-29.6)	5622	347/24445	14.2 (12.8-15.8)
	Missing	613	113/2553	44.3 (36.8-53.2)	893	64/3866	16.6 (13.0-21.2)
Industry group participation	Yes (High SC)	952	108/4059	26.6 (22.0-32.1)	293	6/1304	4.6 (2.1-10.2)
	No (Low SC)	4869	556/20653	26.9 (24.8-29.3)	5510	349/23929	14.6 (13.1-16.2)
	Missing	687	126/2860	44.1 (37.0-52.5)	999	69/4327	15.9 (12.6-20.2)
Volunteer group participation	Yes (High SC)	642	45/2796	16.1 (12.0-21.6)	574	22/2522	8.7 (5.7-13.2)
	No (Low SC)	5122	613/21678	28.3 (26.1-30.6)	5255	331/22829	14.5 (13.0-16.1)
	Missing	744	132/3097	42.6 (35.9-50.5)	973	71/4210	16.9 (13.4-21.3)
Citizen group participation	Yes (High SC)	245	28/1044	26.8 (18.5-38.8)	309	10/1368	7.3 (3.9-13.6)
	No (Low SC)	5465	622/23202	26.8 (24.8-29.0)	5470	346/23752	14.6 (13.1-16.2)
	Missing	798	140/3326	42.1 (35.7-49.7)	1023	68/4441	15.3 (12.1-19.4)
Religious group participation	Yes (High SC)	738	81/3152	25.7 (20.7-31.9)	698	38/3031	12.5 (9.1-17.2)
	No (Low SC)	5021	580/21288	27.2 (25.1-29.6)	5151	319/22391	14.2 (12.8-15.9)
	Missing	749	129/3131	41.2 (34.7-49.0)	953	67/4139	16.2 (12.7-20.6)
Sports group participation	Yes (High SC)	1282	87/5602	15.5 (12.6-19.2)	1152	36/5074	7.1 (5.1-9.8)
	No (Low SC)	4458	564/18776	30.0 (27.7-32.6)	4642	319/20118	15.9 (14.2-17.7)
	Missing	768	139/3193	43.5 (36.9-51.4)	1008	69/4369	15.8 (12.5-20.0)
Neighborhood group participation	Yes (High SC)	3445	384/14716	26.1 (23.6-28.8)	3583	199/15657	12.7 (11.1-14.6)
	No (Low SC)	2531	308/10650	28.9 (25.9-32.3)	2557	170/11058	15.4 (13.2-17.9)
	Missing	532	98/2206	44.4 (36.4-54.1)	662	55/2846	19.3 (14.8-25.2)
Avocation group participation	Yes (High SC)	1592	126/6882	18.3 (15.4-21.8)	2054	75/9046	8.3 (6.6-10.4)
	No (Low SC)	4192	533/17671	30.2 (27.7-32.8)	3819	285/16483	17.3 (15.4-19.4)
	Missing	724	131/3019	43.4 (36.6-51.5)	929	64/4032	15.9 (12.4-20.3)
How often do you meet your friend?	Once or more/month	4360	448/18691	24.0 (21.8-26.3)	5301	296/23134	12.8 (11.4-14.3)
	Several times/year	1077	145/4513	32.1 (27.3-37.8)	610	31/2654	11.7 (8.2-16.6)
	Rarely	752	141/3054	46.2 (39.1-54.5)	529	59/2231	26.4 (20.5-34.1)
	Having no friends	151	24/620	38.7 (25.9-57.7)	125	22/508	43.3 (28.5-65.8)
	Missing	168	32/694	46.1 (32.6-65.2)	237	16/1034	15.5 (9.5-25.3)
Total		6508	790/27572	28.7 (26.7-30.7)	6802	424/29561	14.3 (13.0-15.8)

## Results

The average follow-up period was 4.29 years (SD = 0.75). During 27,571 person-years of follow-up for men and 29,561 person-years of follow-up for women, 790 all-cause deaths in men and 424 in women were observed. The incidence rate per 1000 person-years (IR) of death was 28.7 in men and 14.3 in women. Table 1 and 2 show the distribution of the number of deaths and IR according to covariates and social capital variables. Participants with low social capital in terms of generalised reciprocity and social network tended to have higher IR.

Table 3 shows the univariate and covariates adjusted mortality HRs for the different social capital variables among men. The results of multiple imputation models and non-imputation models were similar, particularly in the univariate models, but the 95%CIs were wider in most of the estimates obtained from the imputation models. In the univariate models using multiple imputation, lower social capital was significantly related to higher mortality in one general trust variable (people try to be helpful: HR = 1.42 (95%CI = 1.01-2.00)), all generalised reciprocity variables (listen to someone's concerns: HR = 1.59 (95%CI = 1.24-2.04); look after someone: HR = 1.49 (95%CI = 1.12-2.00)) and four social network variables (volunteer: HR = 1.78 (95%CI = 1.34-2.37); sports: HR = 1.89 (95%CI = 1.28-2.80); leisure: HR = 1.64 (95%CI = 1.21-2.20); meet friends rarely: HR = 1.99 (95%CI = 1.72-2.31)). When adjusting these models for covariates, only one low social network variable was found to be related to higher mortality (meet friends rarely: HR = 1.30 (95%CI = 1.10-1.53)), while the respective findings for two other social network variables (volunteering and leisure) were marginally not significant

**Table 3:** Univariate and covariate adjusted hazard ratios and 95% confidence intervals for all-cause mortality according to social capital

		Univariate (imputation)			Univariate (non-imputation)			Covariate adjusted (imputation)			Covariate adjusted (non-imputation)		
		HR	95%CI		HR	95%CI		HR	95%CI		HR	95%CI	
General trust													
Generally speaking, would you say that most people can be trusted? (Ref; Yes (High SC))	Depends	0.96	(0.77-1.20)		0.97	(0.80-1.17)		0.90	(0.69-1.16)		1.01	(0.77-1.33)	
	No (Low SC)	1.24	(0.91-1.70)		1.25	(0.96-1.63)		1.01	(0.74-1.36)		0.96	(0.65-1.42)	
Would you say that most of the time people try to be helpful? (Ref; Yes (High SC))	Depends	1.00	(0.84-1.20)		1.01	(0.87-1.16)		0.92	(0.78-1.08)		1.00	(0.86-1.16)	
	No (Low SC)	1.42	(1.01-2.00)	*	1.41	(1.06-1.87)	*	1.20	(0.83-1.74)		1.06	(0.69-1.63)	
Do you think most people would try to take advantage of you if they got a chance? (Ref; Yes (Low SC))	Depends	0.91	(0.63-1.33)		0.92	(0.65-1.30)		1.01	(0.61-1.68)		1.00	(0.57-1.73)	
Social support													
Do you have someone who listens to your concerns and complaints? (Ref; Yes (High SC))	No (Low SC)	1.33	(0.79-2.23)		1.33	(0.80-2.20)		1.09	(0.61-1.95)		1.17	(0.63-2.15)	
Do you have someone who looks after you when you are sick and stay in bed for a few days? (Ref; Yes (High SC))	No (Low SC)	1.06	(0.70-1.61)		1.06	(0.71-1.58)		0.84	(0.58-1.22)		0.87	(0.50-1.52)	
Do you have someone who acknowledges your existence and value? (Ref; Yes (High SC))	No (Low SC)	1.49	(0.99-2.25)		1.48	(0.95-2.30)		1.18	(0.67-2.08)		1.33	(0.70-2.54)	
General reciprocity													
Do you listen to someone's concerns and complaints? (Ref; Yes (High SC))	No (Low SC)	1.59	(1.24-2.04)	*	1.58	(1.31-1.91)	*	1.27	(0.95-1.70)		1.03	(0.70-1.51)	
Do you look after someone when he/she is sick and stays in bed for a few days? (Ref; Yes (High SC))	No (Low SC)	1.49	(1.12-2.00)	*	1.44	(1.13-1.83)	*	1.01	(0.78-1.32)		0.83	(0.70-0.98)	*
Social network													
Political organization or group (Ref; yes)	No (Low SC)	1.14	(0.83-1.56)		1.11	(0.85-1.46)		0.99	(0.71-1.39)		1.11	(0.83-1.49)	
Industrial or trade association (Ref; yes)	No (Low SC)	1.04	(0.72-1.51)		1.02	(0.80-1.29)		0.88	(0.59-1.30)		0.91	(0.62-1.35)	
Volunteer group (Ref; yes)	No (Low SC)	1.78	(1.34-2.37)	*	1.75	(1.39-2.21)	*	1.30	(0.95-1.77)		1.51	(1.19-1.91)	*
Citizen or consumer group (Ref; yes)	No (Low SC)	1.05	(0.59-1.86)		0.98	(0.57-1.68)		0.79	(0.43-1.47)		0.95	(0.37-2.40)	
Religious organization or group (Ref; yes)	No (Low SC)	1.10	(0.77-1.56)		1.11	(0.79-1.56)		1.21	(0.86-1.70)		1.30	(1.06-1.58)	*
Sports group or club (Ref; yes)	No (Low SC)	1.89	(1.28-2.80)	*	1.98	(1.52-2.58)	*	1.32	(0.78-2.21)		1.44	(1.10-1.88)	*
Neighbourhood association / Senior citizen club / Fire-fighting team (Ref; yes)	No (Low SC)	1.16	(0.94-1.42)		1.15	(0.95-1.39)		1.12	(0.90-1.40)		1.20	(0.80-1.81)	
Leisure activity group (Ref; yes)	No (Low SC)	1.64	(1.21-2.20)	*	1.59	(1.40-1.81)	*	1.29	(0.94-1.77)		1.27	(1.04-1.55)	*
How often do you meet your friends? (Ref; Once or more/month)	Several/year	1.26	(0.96-1.67)		1.26	(0.99-1.60)		1.09	(0.77-1.56)		1.06	(0.75-1.48)	
	Rarely	1.99	(1.72-2.31)	*	1.99	(1.75-2.25)	*	1.30	(1.10-1.53)	*	1.38	(1.28-1.49)	*
	Having no friend	1.43	(0.79-2.56)		1.41	(0.90-2.20)		0.77	(0.47-1.26)		0.49	(0.19-1.26)	

Multiple imputation Cox proportional hazard models: the Aichi Gerontological Evaluation Study (AGES), Aichi, Japan, 2003-2008, Men^1^.

^1 ^Adjusted for age, BMI, self-rated health, current illness, smoking history, alcohol consumption, exercise, equivalent income and education.

* Statistically significant variable (p < 0.05).

Table 4 shows the univariate and covariates adjusted mortality HRs for the different social capital variables among women. The results of multiple imputation models and non-imputation models were also similar, particularly in the univariate models, but the 95%CIs were wider in most of the estimates obtained from the imputation models. In the univariate multiple imputation models, lower social capital was significantly related to higher mortality in all generalised reciprocity variables (listen to someone's concerns: HR = 2.31 (95%CI = 1.49-3.58) and look after someone: HR = 1.71 (95%CI = 1.18-2.47)) and four social network variables (sports: HR = 2.32 (95%CI = 1.41-3.82); leisure: HR = 2.24 (95%CI = 1.36-3.68); meet friends rarely: HR = 2.41 (95%CI = 1.31-4.45); having no friends: HR = 3.40 (95%CI = 2.10-5.52)). In the covariate adjusted multiple imputation analysis, only one lower social network response related to higher mortality (having no friends: HR = 1.81 (95%CI = 1.02-3.23)), while findings for one generalised reciprocity variable (listen to someone's concerns) and one social network variable (leisure) were marginally not significant. Interestingly, the response indicating lower social capital in one general trust variable was significantly related to lower mortality (most people cannot be trusted; HR = 0.65 (95%CI = 0.45-0.96)).

**Table 4:** Univariate and covariate adjusted hazard ratios and 95% confidence intervals for all-cause mortality according to social capital

		Univariate (imputation)			Univariate (non-imputation)			Covariate adjusted (imputation)			Covariate adjusted (non-imputation)		
		HR	95%CI		HR	95%CI		HR	95%CI		HR	95%CI	
General trust													
Generally speaking, would you say that most people can be trusted? (Ref; Yes (High SC))	Depends	0.98	(0.67-1.43)		0.99	(0.70-1.42)		0.98	(0.65-1.50)		0.97	(0.79-1.18)	
	No (Low SC)	0.80	(0.55-1.16)		0.80	(0.60-1.05)		0.65	(0.45-0.96)	*	0.62	(0.42-0.93)	*
Would you say that most of the time people try to be helpful? (Ref; Yes (High SC))	Depends	1.26	(0.81-1.95)		1.27	(0.84-1.92)		1.29	(0.72-2.32)		0.95	(0.67-1.34)	
	No (Low SC)	1.69	(1.00-2.87)		1.72	(1.07-2.77)	*	1.49	(0.84-2.64)		1.29	(0.99-1.67)	
Do you think most people would try to take advantage of you if they got a chance? (Ref; Yes (Low SC))	Depends	0.94	(0.54-1.65)		0.94	(0.60-1.48)		1.15	(0.60-2.22)		0.90	(0.41-1.95)	
	No (High SC)	1.10	(0.49-2.49)		1.11	(0.52-2.38)		1.33	(0.49-3.60)		1.21	(0.42-3.50)	
Social support													
Do you have someone who listens to your concerns and complaints? (Ref; Yes (High SC))	No (Low SC)	1.36	(0.80-2.30)		1.36	(0.86-2.14)		1.11	(0.62-1.99)		1.22	(0.57-2.61)	
Do you have someone who looks after you when you are sick and stay in bed for a few days? (Ref; Yes (High SC))	No (Low SC)	0.86	(0.60-1.24)		0.86	(0.59-1.26)		0.84	(0.50-1.43)		0.85	(0.45-1.60)	
Do you have someone who acknowledges your existence and value? (Ref; Yes (High SC))	No (Low SC)	1.31	(0.65-2.66)		1.32	(0.74-2.36)		1.05	(0.56-1.95)		0.91	(0.55-1.51)	
Generalised reciprocity													
Do you listen to someone's concerns and complaints? (Ref; Yes (High SC))	No (Low SC)	2.31	(1.49-3.58)	*	2.38	(1.56-3.63)	*	1.57	(0.96-2.55)		1.40	(0.71-2.77)	
Do you look after someone when he/she is sick and stays in bed for a few days? (Ref; Yes (High SC))	No (Low SC)	1.71	(1.18-2.47)	*	1.72	(1.23-2.39)	*	0.92	(0.63-1.35)		0.73	(0.51-1.05)	
Social network													
Political organization or group (Ref; yes)	No (Low SC)	1.55	(0.58-4.09)		1.55	(0.79-3.02)		1.25	(0.42-3.76)		1.48	(0.63-3.46)	
Industrial or trade association (Ref; yes)	No (Low SC)	2.92	(0.58-14.64)		4.19	(1.46-12.02)	*	1.95	(0.38-9.97)		2.51	(0.77-8.14)	
Volunteer group (Ref; yes)	No (Low SC)	1.76	(0.81-3.83)		1.75	(1.09-2.80)	*	1.06	(0.38-2.95)		0.95	(0.54-1.67)	
Citizen or consumer group (Ref; yes)	No (Low SC)	1.76	(0.45-6.90)		2.07	(1.00-4.30)		1.14	(0.21-6.11)		0.92	(0.56-1.51)	
Religious organization or group (Ref; yes)	No (Low SC)	1.17	(0.77-1.80)		1.19	(0.95-1.50)		1.25	(0.73-2.14)		1.54	(0.84-2.85)	
Sports group or club (Ref; yes)	No (Low SC)	2.32	(1.41-3.82)	*	2.33	(1.72-3.17)	*	1.42	(0.78-2.59)		1.72	(1.11-2.68)	*
Neighbourhood association / Senior citizen club / Fire-fighting team (Ref; yes)	No (Low SC)	1.19	(0.94-1.52)		1.22	(1.03-1.44)	*	1.11	(0.86-1.43)		1.19	(0.95-1.48)	
Leisure activity group (Ref; yes)	No (Low SC)	2.24	(1.36-3.68)	*	2.32	(1.52-3.54)	*	1.54	(0.92-2.57)		1.60	(1.10-2.32)	*
How often do you see your friends? (Ref; Once or more/month)	Several/year	1.00	(0.64-1.58)		0.99	(0.68-1.42)		0.92	(0.58-1.46)		0.87	(0.40-1.87)	
	Rarely	2.41	(1.31-4.45)	*	2.44	(1.40-4.26)	*	1.64	(0.90-2.98)		1.62	(0.82-3.22)	
	Having no friend	3.40	(2.10-5.52)	*	3.55	(2.37-5.32)	*	1.81	(1.02-3.23)	*	2.10	(0.71-6.26)	

Multiple imputation Cox proportional hazard models: the Aichi Gerontological Evaluation Study (AGES), Aichi, Japan, 2003-2008, Women^1^.

^1 ^Adjusted for age, BMI, self-rated health, current illness, smoking history, alcohol consumption, exercise, equivalent income and education.

* Statistically significant variable (p < 0.05).

## Discussion

To the best of our knowledge, this study is the first prospective cohort study to assess the relationships between various social capital measures and mortality. In addition, this is the first cohort study on the relationship between social capital and mortality in a non-Western country. The present study showed that the structural social capital variable (friendship network) was a good predictor for all-cause mortality among older Japanese. Among men, it was the frequency of meetings with friends that was important, with those meeting their friends rarely having higher mortality, while was it was the lack of friends that was indicative of higher mortality among women. In addition, low general trust was related to lower mortality among women, suggesting that general trust has a different meaning among older Japanese women than among men.

Our results suggested the existence of culture differences in the association between trust and health. In addition, it is possible that the specific questions used to measure trust may also play a role. In our study, the question about general trust ("Generally speaking, would you say that most people can be trusted?") measures the trust for strangers, not group members [28]. In Japan, a relatively collectivist society with intense group ties, human relations are based on mutual assurance between group members rather than mutual trust between out-group members [11,13]. The systems of mutual assurance, monitoring and sanctioning, within groups make the Japanese society safe and stable though closed [11,13]. In such a society with strong ties, people can relatively easily obtain social support [29]. Though Japanese society is gradually changing recently because of globalisation, older people have lived in this traditional type of society for a long time throughout their life-course. Our results could suggest that Japanese older women who did not trust others would adapt well to the collectivist society with intense group ties and benefit from the society. In this Japanese older generation, men tended to work outside while women were predominantly housewives, therefore, men had to communicate with out-group members during their work and this may have contributed to developing their general trust towards strangers. In contrast, lower general trust measured by the question "Would you say that most of the time people try to be helpful?" tended to be associated with higher mortality among both men and women. This may be a more applicable question for measuring general trust among older Japanese, as this cohort may refer to their group members as "people" when answering this question. Previous cohort studies have not used general questions on trusting people to measure social capital. In Finland, a prospective study determined the beneficial effect of trust on all-cause mortality among women aged 30-99 years, not men [2]. Their measure of trust was based on the number of and trust in close friends. In this study, we did not use the factor/principal component analysis to check the association between detailed, not combined, social capital variables and mortality. As the results, various social capital variables were included into the models and different association of trust questions on mortality were shown though this method had the possibility of a type 1 error. Further prospective research assessing trust and mortality with considering various culture backgrounds is needed.

Our results are partially consistent with those of previous studies. Social network and social support are positively associated with health. A meta-analysis of social relationships and mortality determined that strong structural social relationships (social network) and functional social relationships (social support) increased the likelihood of survival [30]. In line with this, our study showed significant associations between friendship network and mortality (meet friends rarely for men; HR = 1.30 (95%CI = 1.10-1.53), having no friends for women; HR = 1.81 (95%CI = 1.02-3.23)); however, social support was not significantly associated with mortality. Although we considered diagnosed diseases and excluded from our study people with limitations in basic activities of daily living, it is possible that people included in the study may have had latent fatal diseases and consequently needed some help; this may have affected our results about social support and mortality. The concept of generalised reciprocity is based on the assumption that when people provide resources, good turns will be repaid at some unspecified time in the future, perhaps even by an stranger [31]. It does not entail tit-for-tat calculations in which individuals can be sure that a good turn will be repaid quickly and automatically [31]. Therefore, we used variables about the provision of social support as generalised reciprocity variables. In our study, generalised reciprocity showed marginal though non-significant association with mortality (HR = 1.27 (95%CI = 0.95-1.70) for men and HR = 1.57 (95%CI = 0.96-2.55) for women). A prospective cohort study in Finland showed that leisure participation was significantly though marginally associated with reduced all-cause mortality (HR = 0.94 (95%CI = 0.89-1.00) for men and HR = 0.96 (95%CI = 0.91-1.00) for women). In our study, covariate adjusted HRs of leisure participation were again marginal, though non-significant (HR = 1.29 (95%CI = 0.94-1.77) for men and HR = 1.54 (95%CI = 0.92-2.57) for women). The meaning of volunteering varies in the societies of different cultures [8] and relationships between volunteering and mortality are not consistent across studies [3-5]. In our study, covariate adjusted HR of volunteer participation was marginal though non-significant for men (HR = 1.30 (95%CI = 0.95-1.77)) and non-significant for women (HR = 1.06 (95%CI = 0.38-2.95)).

There are several plausible pathways linking social capital to health [32]. At first, social capital may affect individual health by influencing health-related behaviours through promotion of more rapid diffusion of health information and by exerting social control over deviant health-related behaviours [32]. Second, higher social capital may promote health by increasing access to local services and amenities [32]. Good access to service such as transportation, clinics and community health centres could improve health. Third, there are associations between social capital and psychological distress [33,34]. Social networks and social support can buffer the negative effects of life events on mental health [34]. Fourth, the communities with higher social capital produce more egalitarian patterns of political participation that result in the implementation of policies which ensure the security of all its members [32].

The results of this study have important public health implications. Among older Japanese, structural social capital variable related to friendship network were found to be significantly associated with mortality regardless of various covariates. This result suggests the possibility that public investment to promote social network may reduce the mortality among older people.

Our study has a number of limitations and strengths. The follow-up period (4.29 years) was relatively short. There was a potential bias caused by latent fatal disease though we considered diagnosed diseases and limitations in basic activities of daily living at baseline. In addition, the response rate was 50.4%; therefore, the results may have been affected by selection bias. Hanibuchi et al. previously conducted ecological analysis that assessed associations between community-level social capital and response rate using another data set from the AGES project and found that higher response rates were significantly associated with higher social capital [35]. Therefore, respondents of this study might have higher social capital than non-respondents. Although our results showed significant effects of some dimensions of social capital on all-cause mortality, this low response rate might have attenuated that association. In addition, compared with government data, our study respondents tended to be younger. It could be argued that healthier and younger people tend to respond to our questionnaire while people with higher risks of mortality tend to not participate. If so, this might have contributed to an underestimation of the association between poor social capital and mortality. As a strength, the present study used various social capital variables. Although the validity and reliability of the social capital variables were not been directly examined, a previous study using AGES project data checked the association between social capital variables based on our survey and voting rate, rate of volunteer registration and rate of social participation based on public social survey data [35]. Mean response of trust variable measured by our survey significantly associated with rate of volunteer registration in each community. Similarly, social network variables were significantly associated with voting rate.

## Conclusions

In conclusion, friendship network, a measure of individual social capital, was a good predictor for all-cause mortality among older Japanese. In addition, low general trust was related to lower mortality among women. Further studies examining the different effect of social capital between Western and non-Western countries are needed.

## Competing interests

The authors declare that they have no competing interests.

## Authors' contributions

JA had the idea for the study, participated in its design, performed the statistical analysis and drafted the manuscript as a principal author. KK helped develop the idea of the study, participated in acquiring the data and with design, and edited the manuscript. HH participated in acquiring the data and critically revising the manuscript. SVS participated in design of study, data analysis and critically revised the manuscript. CM, NK, YI, KS and KO participated in design of study and critically revised the manuscript. All authors read and approved the final manuscript.

## Pre-publication history

The pre-publication history for this paper can be accessed here:

http://www.biomedcentral.com/1471-2458/11/499/prepub
